# Pringle-Shaped Mesoporous Li_4_Ti_5_O_12_/C Composite with Enhanced Rate Performance for Lithium-Ion Batteries

**DOI:** 10.3390/molecules31101671

**Published:** 2026-05-15

**Authors:** Yanfang Huo, Jingxiao Tang, Yanqing Huo, Min Wang, Likun Han, Jinhua Liu

**Affiliations:** 1 College of Chemistry & Chemical and Environmental Engineering, Weifang University, Weifang 261061, China; 2 College of Advanced Agriculture and Life Sciences, Weifang University, Weifang 261061, China

**Keywords:** spinel lithium titanate, carbon coating, synergistic effect, Li-ion batteries

## Abstract

Despite exhibiting exceptional structural stability, spinel lithium titanate (Li_4_Ti_5_O_12_, LTO) suffers from inherent limitations in both electronic conductivity and ionic diffusion kinetics, limiting its high-rate application. In this study, pringle-shaped Li_4_Ti_5_O_12_/carbon (LTO/C) composite was synthesized using low-cost sucrose as the organic carbon source, using a facile hydrothermal-calcination method. Each pringle-shaped nanosheet was composed of Li_4_Ti_5_O_12_ nanoparticles that are 15 nm in size, which can shorten lithium-ion diffusion distances as well as better the contact between electrolyte and active materials. Meanwhile, the uniform carbon cladding improves the material’s electronic conductivity. Owing to the synergistic effects between the mesoporous LTO nanosheets and carbon coating, LTO/C-6.31 wt% presented remarkable rate capability and cycling stability, delivering 145.5 mAh g^−1^ at 20 C over 1000 cycles with 93.93% capacity retention. This work demonstrates an effective synthesis route to developing high-rate capability and long-cycle-life anode materials for LIBs.

## 1. Introduction

Energy is a crucial global issue. With the rapid proliferation of communication devices, health monitoring devices, and most notably, various types of electrical vehicles (HEVs, BEVs, and PHEVs), Lithium-Ion Batteries (LIBs) have garnered extensive and intensive research interest owing to their excellent safety, long cycle life, and environmental sustainability [1,2,3,4]. It is well established that the enhancement of safety and cycling stability in battery systems is heavily dependent on the performance of anode materials. Thanks to their low cost, superior electrical conductivity, and abundant raw material sources, carbon-based materials are commonly adopted as anodes for lithium-ion batteries. However, their practical application faces two critical challenges: (1) Structural degradation caused by repetitive volume expansion/contraction throughout the course of lithiation/delithiation cycling ultimately results in capacity fading; (2) The low lithium insertion potential of carbon anodes facilitates lithium dendrite formation, posing safety risks. Such inherent limitations of conventional carbon electrodes significantly impair both battery lifespan and operational safety [5,6].

Compared to the conventional graphite/carbon-based anodes for LIBs, spinel lithium titanate (Li_4_Ti_5_O_12_, LTO) exhibits certain advantages. On the one hand, the relatively high potential plateau (1.55 V vs. Li/Li^+^) enhances the safety of LIBs by avoiding two issues: (i) The formation of a solid electrolyte interphase (SEI) film (usually occurring below 1.0 V vs. Li/Li^+^) on the surface of an electrode, and (ii) lithium dendrite during fast charging [7,8,9]. On the other hand, LTO undergoes a negligible volume deformation during Li^+^ extraction/intercalation, thereby enabling a superior cycling performance in LIBs [10,11,12]. However, inherent drawbacks exist in any material, and LTO is no exception. The major challenge of LTO materials currently lies in their extremely low electronic conductivity (~10^−13^ S cm^−1^) and the intrinsic low diffusion coefficient of Li^+^ (10^−9^ to 10^−13^ cm^2^ s^−1^) that results in severe polarization, thus greatly limiting its reversible capacity at high rates [13,14,15].

To address the drawbacks mentioned, various methods have been implemented to enhance rate performance, specific capacity, and cycle stability, such as morphology control [16,17,18,19], surface modification [20,21,22], and element doping [7,23,24,25] in the last decade. To enhance rate performance, modification with carbon materials is the most impactful strategy. Hu et al. [22] fabricated a carbon-coated lithium titanate (CC-LTO) featuring a nanosheet structure via a solvothermal reaction followed by calcination. The CC-LTO exhibited a specific discharge capacity of 153.9 mAh g^−1^ (80 C) and 137.9 mAh g^−1^ (160 C), along with excellent cycling stability (77.2% retention after 10,000 cycles at 10 C). Li et al. [26] successfully synthesized a core-shell structured LTO@C particle (~320 nm, carbon shell ~10 nm), demonstrating an impressive 90.45% capacity retention even at high rates of 20 C after 200 cycles. An ideal anode material should possess the following three characteristics: (1) excellent electronic conductivity; (2) a hierarchical structure composed of small particles that facilitate Li^+^ diffusion and electrolyte infiltration; (3) large reversible Gibbs energy during the reaction with Li^+^, which can reduce the polarization-induced energy loss and ensure a high capacity [9,13,27].

In this study, we successfully synthesized pringle-shaped mesoporous Li_4_Ti_5_O_12_/carbon composite (LTO/C) using a hydrothermal-calcination method. The amorphous carbon layer serves to enhance the electronic conductivity of LTO and facilitate the preparation of mesoporous LTO with smaller particles. This structure effectively shortens the Li^+^ diffusion pathway, thereby promoting Li^+^ transport and facilitating the immersion of the electrolyte. Thus, achieving an appropriate balance between carbon and LTO is crucial, as it greatly enhances conductivity and discharge capacity. The findings indicate that a carbon weight ratio of 6.31 wt% yields the best electrochemical performance, demonstrating its potential as a highly promising anode material for lithium-ion batteries. The as-prepared LTO/C-6.31 wt% composite exhibits a high reversible-specific capacity of 145.5 mAh g^−1^ (93.93% capacity retention) at 20 C after 1000 cycles.

## 2. Experimental Section

### 2.1. Materials Preparation

The carbon-coated LTO (LTO/C) composites and pure LTO were synthesized using a modified hydrothermal process [28]. All chemical reagents were directly used without further purification. Firstly, LiOH·H_2_O 420 mg and TBT 3.4 mL were dissolved in 50 mL ethanol within a dry flask and then stirred for 12 h at 25 °C (designated Solution A). After that, Solution B was obtained by dissolving 120 mg sucrose in 50 mL of deionized water. Subsequently, solution B was added dropwise into solution A under continuous stirring for 30 min. The prepared homogeneous solution was transferred into a 150 mL Teflon-lined stainless-steel autoclave and heated at 180 °C for 36 h for the hydrothermal reaction. After being cooled to room temperature, the precursor was washed with ethanol, and vacuum dried at 80 °C for 12 h. The dried powder precursor was ground and annealed at 600 °C for 6 h in an Ar atmosphere with a ramp of 10 °C min^−1^, in order to obtain Li_4_Ti_5_O_12_/C. To determine the suitable carbon content, composites were prepared with varying sucrose masses (0 mg, 160 mg, and 200 mg) while maintaining all other experimental parameters at a constant.

### 2.2. Materials Characterization

X-ray diffraction (XRD, D/MAX-2500, Rigaku, Tokyo, Japan) was used to analyze the crystal structures of all composites. Thermogravimetric analysis (TGA, NETZSCH STA 449 F3 apparatus, Netzsch, Selb, Germany) was employed to determine the carbon content. X-ray photoelectron spectroscopy (XPS, PHI1600, ULVAC-PHI, Chigasaki, Kanagawa, Japan) was used to probe the elements and valences on the surface of the samples. The morphological features and particle sizes were examined by scanning electron microscopy (SEM, S4800, Hitachi, Tokyo, Japan) and transmission electron microscopy (TEM, Tecnai G2 F20, FEI, Hillsboro, OR, USA), while high resolution transmission electron microscopy (HRTEM, Tecnai G2 F20, FEI, Hillsboro, OR, USA) recorded the lattice structure. The nitrogen adsorption-desorption isotherms were obtained by Brunauer-Emmett-Teller (BET, KUBO-X1000, Builder Electronics, Beijing, China). 

### 2.3. Li-Ion Cell Assembly and Electrochemical Measurements

A homogeneous slurry was prepared by mixing the synthesized electrode materials, PVDF binder, and acetylene black in a weight ratio of 8:1:1 with NMP. Subsequently, the homogenous slurry was then coated on the copper foil, which was dried at 120 °C under a vacuum for 12 h. The detailed electrode parameters are provided as follows: The active material mass loading is ~1.0 mg cm^−2^, the electrode thickness is ~40 μm. Owing to the unique pringle-shaped and highly porous structure of the prepared LTO material, the electrode presents a relatively low compaction density of ~0.25 g cm^−3^, which is obviously lower than that of commercial LTO materials. The abundant internal pores are conducive to electrolyte infiltration and shorten the ion diffusion path, thus improving the rate capability. Nevertheless, the high porosity inevitably reduces the volumetric energy density of the electrode. The CR2032 coin-type half-cell consisted of a positive electrode, a porous polypropylene separator, a lithium foil counter electrode, and 1M LiPF_6_ in EC:DMC (1:1 in volume) as the electrolyte, assembled in an argon-filled glovebox with a water content of less than 0.01 ppm. The LAND CT-2001A system(CT2001A, Wuhan Land Electronics Co., Ltd., Wuhan, China) was used to perform the galvanostatic cyclic charge–discharge tests within a voltage window of 1.0–2.5 V. Cyclic voltammetry (1.0–2.5 V at variable scan rates) and electrochemical impedance spectroscopy (100 kHz–0.01 Hz) were carried out on a CHI760E electrochemical workstation(Chenhua CHI760E, Shanghai Chenhua International Trade Co., Ltd., Shanghai, China).

## 3. Results and Discussions

### 3.1. Characterization

**Figure 1:**
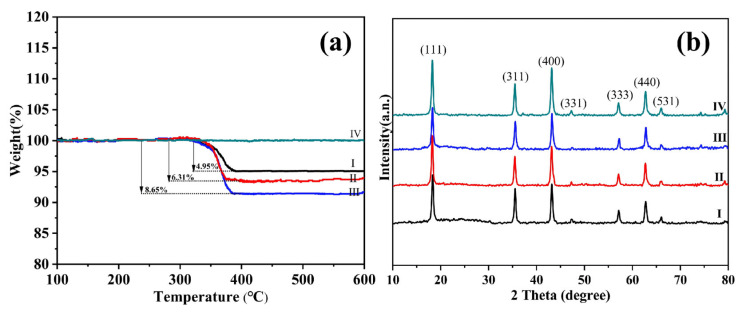
(**a**) TG curves (air atmosphere) of LTO (IV) and LTO/C with 120, 160, and 200 mg sucrose (samples I–III); (**b**) XRD patterns of pure LTO (IV) and LTO/C with varying carbon weight ratios of 4.95%, 6.31%, and 8.65% (samples I–III).

As illustrated in Figure 1a, thermogravimetric analysis (TGA) was conducted to evaluate the carbon contents in the fabricated Li_4_Ti_5_O_12_/C composites under air atmosphere. The results show that the carbon decomposes thermally at approximately 300–400 °C and the mass loss corresponds to the carbon coating content of the composite. Thus, based on the result, it can be calculated that the carbon coating contents of composites are about 4.95 wt% (I), 6.31 wt% (II), and 8.65 wt% (III) respectively. XRD patterns of LTO/C composites with carbon contents of 4.95 wt%, 6.31 wt%, and 8.65 wt% (patterns I, II, and III, respectively) and pure LTO are presented in Figure 1b. Seven distinct diffraction peaks of all LTO/C composites at 2θ of 18.3° (111), 35.6° (311), 43.2° (400), 47.3° (331), 57.3° (333), 62.8° (440), and 66° (531) match well with spinel Li_4_Ti_5_O_12_ (JCPDS Card No. 49-0207). This confirms that carbon inclusion does not alter the intrinsic lattice structure of LTO, maintaining its characteristic crystal structure. Crucially, no characteristic peak of carbon was observed in the LTO/C patterns, attributable to the low carbon content and its amorphous characteristics [26].

**Figure 2:**
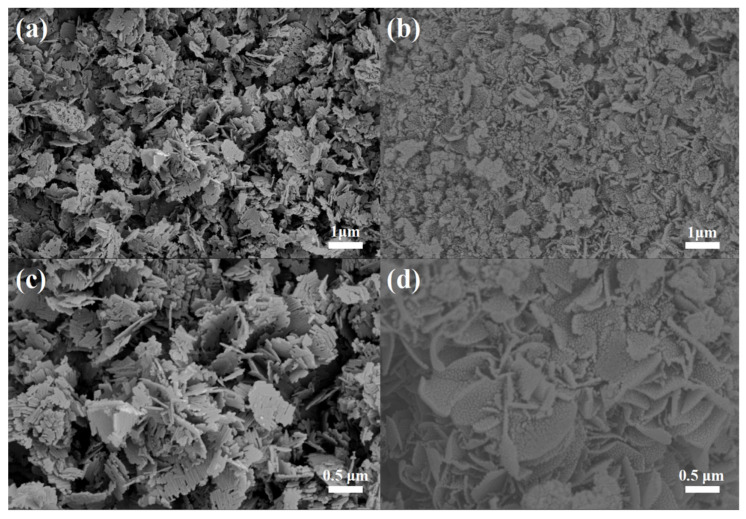
SEM images of LTO (**a**,**c**) and LTO/C-6.31 wt% (**b**,**d**).

The morphologies and microstructures of LTO and LTO/C-6.31 wt% were examined by SEM at different magnifications. The SEM images of LTO (Figure 2a,c) reveal that the smooth nanosheets comprise LTO particles, exhibiting dimensions of approximately 500 nm in length and 300 nm in width. As depicted in Figure 2b,d, due to the inclusion of carbon, LTO/C-6.31 wt% composite consists of rough pringle-shaped nanosheets, which can not only facilitate the insertion and extraction of Li^+^ but also prohibit LTO nanosheets from aggregating. Meanwhile, the uniform carbon shell coating on an LTO establishes a continuous conductive network while eliminating carbon-deficient blind spots inherent to non-uniform coatings, thereby synergistically enhancing the material’s electron collection efficiency and overall conductivity. More importantly, each LTO nanosheet of composite comprises several interconnected nanoparticles, approximately 15 nm in diameter, with some spacing between them. This structural feature significantly enhances the efficiency of electrolyte penetration, thereby accelerating Li^+^/electron transfer at the electrode–electrolyte interface. Thanks to the unique structure of LTO/C-6.31 wt%, the high conductivity of carbon enhances both electronic and ionic transport within the LTO. Meanwhile, the carbon content is optimized to balance electron and ion migration kinetics [29].

**Figure 3:**
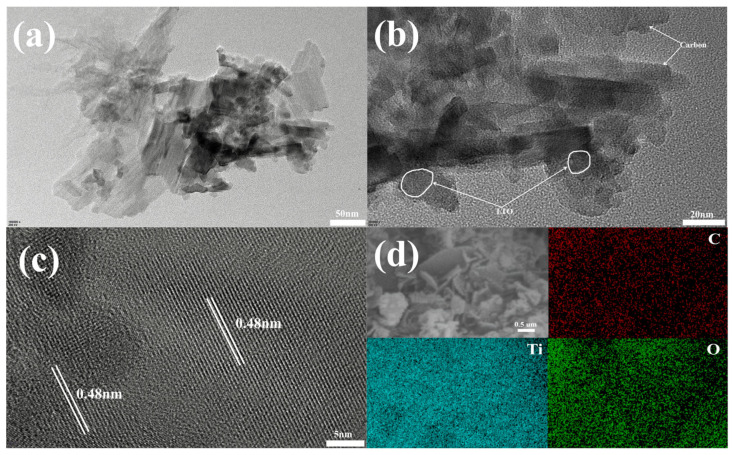
TEM (**a**) and HRTEM (**b**,**c**) images of LTO/C-6.31 wt%. EDX mapping image of LTO/C-6.31 wt% (**d**).

TEM was employed to further analyze the detailed morphology and microstructure of LTO/C-6.31 wt%. As illustrated in Figure 3a, grains with a diameter of 10–15 nm are interspersed across the LTO nanosheets. To identify these small grains, magnified views were observed, as shown in Figure 3b,c. The small grains were identified by their characteristic lattice fringes with a spacing of 0.48 nm, which precisely matched the (111) planes of spinel LTO. Furthermore, a small amount of amorphous carbon is distributed around the edges and on the surface of the LTO. Furthermore, EDX mapping was conducted to probe the surface properties of LTO/C-6.31 wt%, as depicted in Figure 3d. It should be noted that lithium (Li) is an ultra-light element and cannot be detected by conventional EDX. The EDX mapping clearly shows that no detectable impurity elements exist except C, Ti, and O. Moreover, the homogeneous spatial distribution of these elements provides strong evidence for a uniform carbon coating on LTO.

**Figure 4:**
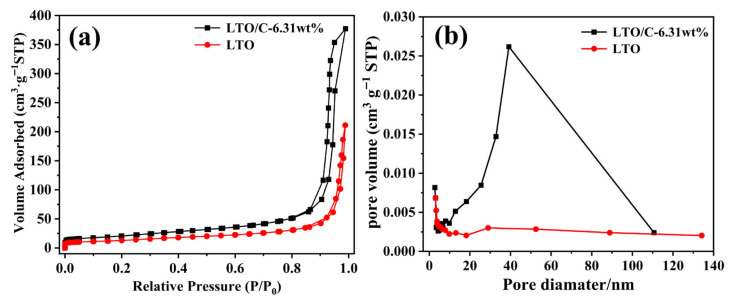
Nitrogen adsorption–desorption isotherms (**a**) and BJH pore-size distributions (**b**) of LTO and LTO/C-6.31 wt%.

N_2_ adsorption–desorption measurements were performed to investigate the effect of carbon coating on the pore structure of LTO materials, presented in Figure 4. Both samples display typical type IV isotherms with distinct hysteresis loops, indicating a mostly mesoporous structure. LTO/C-6.31 wt% shows a significantly higher N_2_ uptake compared with LTO, suggesting that carbon coating substantially increases both the pore volume and specific surface area. According to BET analysis, LTO has a specific surface area of 48.74 m^2^ g^−1^ and an average pore size of 26.82 nm, whereas LTO/C-6.31 wt% exhibits an increased specific surface area of 74.39 m^2^ g^−1^, a larger average pore size of 31.38 nm, and an enhanced pore volume from 0.327 to 0.584 cm^3^ g^−1^. The pore size distribution curve (Figure 4b) reveals a prominent mesoporous peak for LTO/C-6.31 wt% in the range of 30–40 nm. This indicates that the combination of pore generation from the pyrolysis of carbon source and particle isolation during carbon coating successfully creates well-developed hierarchical mesoporous channels. Such a hierarchical mesoporous structure, characterized by simultaneous increases in specific surface area and average pore size, provides favorable conditions for electrolyte infiltration and rapid Li^+^ transport, which is beneficial for improving the rate and cycle performance of the material.

Results of SEM, TEM, and BET analyses conclusively demonstrate that the synergistic effect of LTO and C leads to the formation of pringle-shaped mesoporous Li_4_Ti_5_O_12_/C composite, which can significantly contribute to the rapid transmission of Li^+^ at the electrode/electrolyte interface.

**Figure 5:**
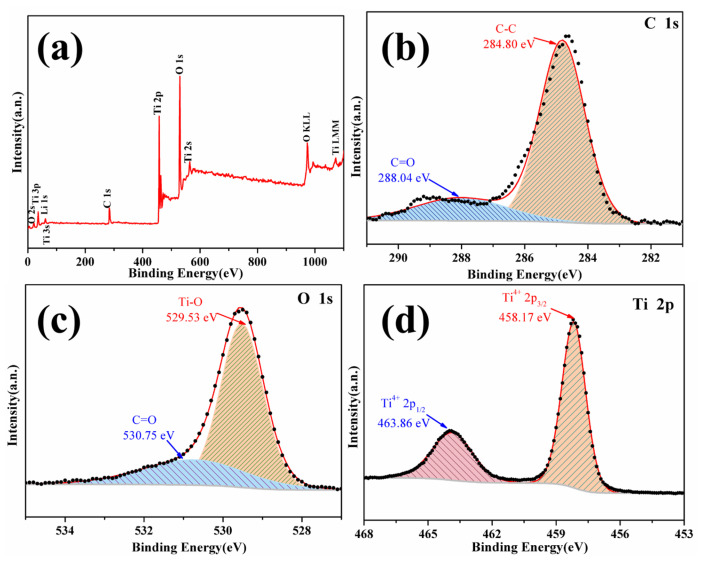
XPS spectrum of LTO/C-6.31 wt%. Survey spectra (**a**), C 1s region (**b**), O 1s region (**c**), Ti 2p region (**d**).

XPS was employed to elaborate the elemental composition and chemical bonding state of LTO/C-6.31 wt% surface. The full XPS spectrum (Figure 5a) confirms the presence of Li, C, O, and Ti elements, consistent with EDX results. The C1s spectrum (Figure 5b) of LTO/C-6.31 wt% exhibits two characteristic peaks at 284.8 and 288.04 eV, corresponding to C-C and C=O bonds. In the O 1s region (Figure 5c), two distinct peaks are observed at 529.53 and 530.75 eV, corresponding to Ti-O and C=O species. The deconvoluted Ti 2p spectrum (Figure 5d) reveals two peaks at 458.17 eV (Ti 2p_3/2_) and 463.86 eV (Ti 2p_1/2_), both corresponding to Ti^4+^, indicating that the chemical states of Ti in LTO/C-6.31 wt% are identical to those of LTO. No other Ti valence states are detected on the LTO/C-6.31 wt% surface, confirming its high phase purity.

### 3.2. Electrochemical Measurement

**Figure 6:**
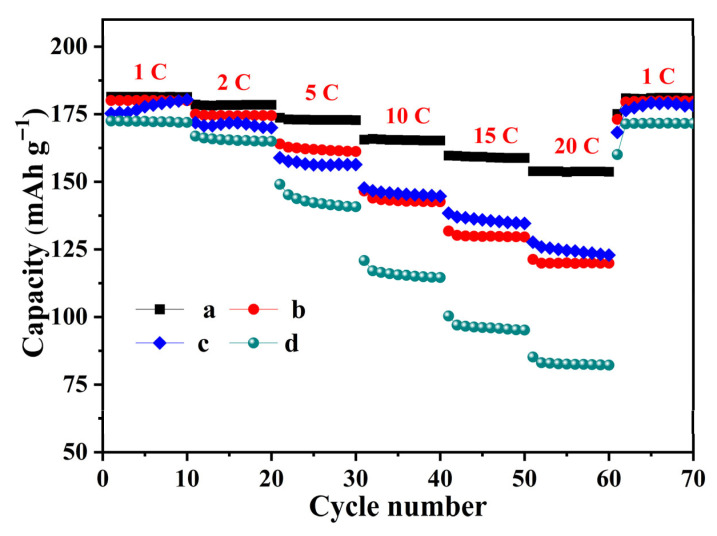
Discharge capacity of LTO/C composites (6.31 wt%, 4.95 wt%, 8.65 wt%; a, b, c, respectively) and LTO (d) at different rates.

Electrochemical performances were evaluated in half-cells employing lithium metal as the counter electrode. The theoretical specific capacity of Li_4_Ti_5_O_12_ is 175 mAh g^−1^. Accordingly, 1 C is defined as 175 mA g^−1^ based on theoretical capacity, which serves as the conversion basis for all rate currents in this work. The rate capacity of Li-ion batteries assembled with LTO/C composite (6.31 wt%, 4.95 wt%, 8.65 wt%) and bare LTO were tested at 1–20 C with a voltage window of 1.0–2.5 V (vs. Li/Li^+^), shown in Figure 6. 10 cycles were performed at each rate before switching back to 1 C. LTO/C-6.31 wt% exhibits superior rate capacity, especially at higher current densities. At 1 C, its discharge capacity (181.5 mAh g^−1^) is slightly higher than those of LTO/C-4.95 wt% (180.1 mAh g^−1^), LTO/C-8.65 wt% (175.3 mAh g^−1^) and pure LTO (172.5 mAh g^−1^). At 20 C, the corresponding capacities decrease to 154.9, 121.3, 127.7, and 85.2 mAh g^−1^, corresponding to capacity retentions of 85.1%, 67.4%, 72.8% and 49.4%. Moreover, upon returning to 1 C, its capacity fully recovers to the initial value, demonstrating excellent cycle reversibility. The superior electrochemical performance of LTO/C-6.31 wt% is ascribed to the unique pringle-shaped structure and mesoporous nature, facilitating effective contact between active materials and electrolytes [30,31]. Meanwhile, the uniform carbon coating and shortened Li^+^ diffusion paths synergistically boost the Li^+^ diffusion kinetics and electronic conductivity of the composite.

Notably, the discharge capacity of LTO/C-6.31 wt% (181.5 mAh g^−1^, 1 C) exceeds the theoretical capacity of LTO (175 mAh g^−1^, 1 C). This can be rationally attributed to two synergistic effects. First, the uniform amorphous carbon shell improves the electron collection and conductivity, establishing an effective conductive network that avoids “blind spots” and thereby improves the overall electron transport of the composite. Second, the uniformly coated amorphous carbon layer contributes additional capacity during electrochemical cycling [26].

It is noted that the thickness and uniformity of the carbon layer significantly affects the electrochemical performance of the composite. An insufficient carbon source fails to form a conformal coating on the LTO particle surface, compromising its electronic conductivity. Conversely, excessive carbon source leads to an overly thick carbon layer that physically impedes Li^+^ diffusion [22]. Consequently, LTO/C-6.31 wt% demonstrated significantly enhanced rate capability compared to counterparts containing 4.95 wt% and 8.65 wt% carbon.

**Figure 7:**
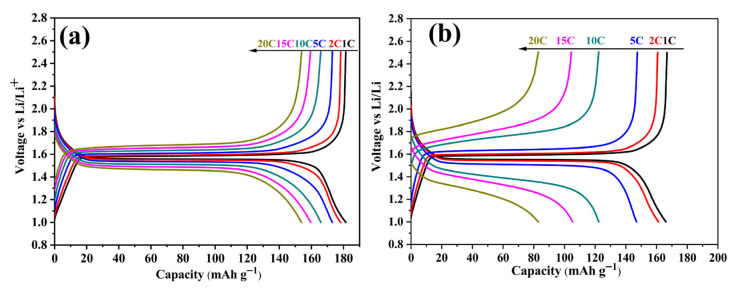
The charge/discharge curves of LTO/C-6.31 wt% (**a**) and LTO (**b**) from 1 C to 20 C.

The galvanostatic charge/discharge curves of LTO/C-6.31 wt% and LTO are displayed in Figure 7a,b. Notably, the flat charge–discharge voltage platforms appear at approximately 1.55 V under low rates, which correspond to the typical two-phases transformation between Li_4_Ti_5_O_12_ and Li_7_Ti_5_O_12_ (Li_4_Ti_5_O_12_ + 3Li^+^ + 3e^−^ ⇌ Li_7_Ti_5_O_12_) [32]. As current density increases, the length of charge–discharge plateau gradually shortens, while the potential gap between the charge and discharge plateaus widens, indicating the increased polarization of electrode material. In contrast, LTO/C-6.31 wt% demonstrates a notably prolonged Li^+^ insertion/extraction at high rates, signifying the enhanced Li^+^ transport capacity and implying the existence of supplementary Li storage mechanisms, which synergistically improves both specific capacity and high-rate performance.

**Figure 8:**
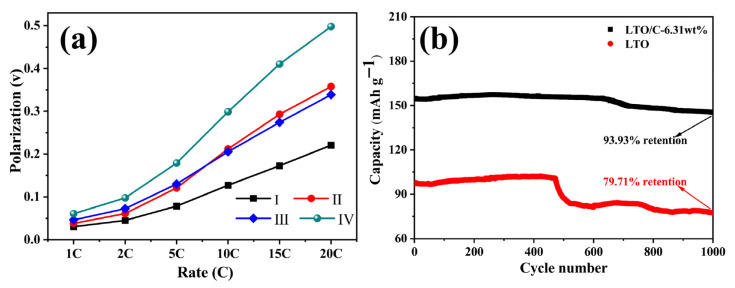
(**a**) The potential gaps of LTO/C (6.31 wt%, 4.95 wt%, 8.65 wt%; I, II and III, respectively) and LTO (IV). (**b**) Cycling performance of LTO/C-6.31 wt% and LTO at 20 C.

Furthermore, the potential gaps of each sample at various current rate (tested at a 50% depth of charge–discharge) are depicted in Figure 8a. The potential difference (ΔU) of the LTO increases sharply with escalating current density, reaching approximately 0.498 V at 20 C, and that of the LTO/C-6.31 wt% exhibits only a gradual rise, maintaining a value of merely about 0.2207 V at the same rate. This indicates that LTO/C-6.31 wt% exhibits the minimal degree of polarization, reflecting its superior electrochemical reversibility and favorable kinetic properties. Such remarkable rate capability can be ascribed to two primary factors: (i) the improved electrical contact between Li_4_Ti_5_O_12_ particles through the three-dimensional conductive network constructed by carbon; (ii) the synergistic effect of nanosheet morphology and porous structure can accelerate the transfer of Li^+^/electron transport kinetics between the active material and the electrolyte, resulting in low polarization.

Encouraged by the superior rate capability performance, the long-term cycling performances were examined at the rate of 20 C within a voltage range of 1.0–2.5 V, as presented in Figure 8b. The result indicates that LTO/C-6.31 wt% exhibits excellent cycling stability, possessing a higher initial discharge capacity (154.9 mAh g^−1^) compared to that of LTO (97.1 mAh g^−1^). The capacity of LTO/C-6.31 wt% maintains 145.5 mAh g^−1^ (93.93% retention), whereas pure LTO delivers only 77.4 mAh g^−1^ (79.71% retention), after 1000 cycles. The enhanced cycling stability can be ascribed to two primary factors. Firstly, the interspace within the carbon component combined with the crooked mesoporous of LTO can enhance the transportation of Li^+^ and the infiltration of the electrolyte, promoting rapid electrode reaction kinetics and consequently diminishing the overpotential. Secondly, mesoporous LTO and carbon synergistically create an electronic conduction network between otherwise insulated LTO particles, thus boosting electrochemical performance. As summarized in Table 1, LTO/C-6.31 wt% also demonstrates superior rate and cycling performance compared to other reported LTO-based composites.

**Figure 9:**
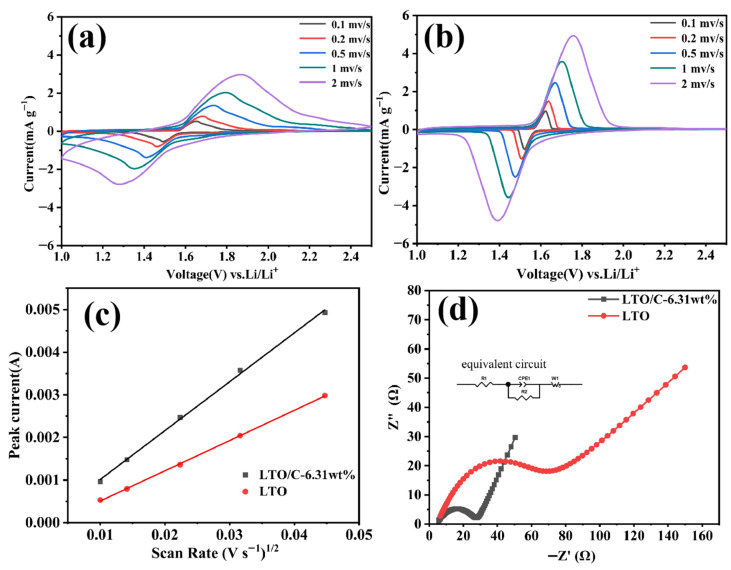
CV curves of LTO (**a**) and LTO/C-6.31 wt% (**b**); (**c**) Peak current (Ip) as a function of square root of scan rate v^1/2^; (**d**) EIS of LTO and LTO/C-6.31 wt%, Inset: equivalent circuit model.

To delve deeper into the electrochemical kinetic behaviors of LTO/C-6.31 wt% and LTO, cyclic voltammetry (CV) tests were conducted within the window of 1.0–2.5 V. The outcomes are depicted in Figure 9a,b. Notably, all CV profiles present a well-defined pair of reduction/oxidation peaks, attributing to the redox reaction involving the Ti^4+^/Ti^3+^ couple (Li_7_Ti_5_O_12_↔Li_4_Ti_5_O_12_ + 3Li^+^ + 3e^−^) [32]. Apparently, LTO/C-6.31 wt% composite exhibited larger total CV peak areas (sum of oxidation and reduction peak areas) than pure LTO at an identical scan rate, indicating that the high amount of electric charges were transferred, being consistent with the fact that the discharge capacity of the composite was notably higher than that of pure LTO. Evidently, the LTO/C-6.31 wt% electrode exhibits not only a markedly higher peak current density (Ip) but also a smaller voltage gap (|ΔEP|) between its redox peaks, compared with the pure LTO electrode. Due to the synergistic effect of the ultrathin nanosheet structure and carbon capping layer, LTO/C-6.31 wt% composite demonstrates the most pronounced redox peak, the largest redox peak current, and the minimal redox peak potential difference, suggesting its accelerated electrochemical reaction kinetics. Figure 9c shows a good linear relationship between peak current and square root of scan rate, indicating that the electrode reaction process is diffusion-controlled [33]. Given that the diffusion coefficient is directly proportional to the slope of this linear fit, the steeper slope observed for the LTO/C-6.31 wt% specimen indicates enhanced Li^+^ diffusion kinetics and, consequently, an improved rate capability. For a diffusion-controlled process, the lithium-ion diffusion coefficient follows the Randles–Sevcik equation: *I_p_ =* 269,000*n*^3/2^*ACD*^1/2^*v*^1/2^, where n is the number of electrons transferred during Li intercalation, A is the surface area of the LTO anode, and C is the concentration of Li^+^ in the LTO. Based on this equation, the Li diffusion coefficient of LTO/C-6.31 wt% is calculated to be 1.41 × 10^−7^ cm^2^ s^−1^, approximately three times that of LTO (5.38 × 10^−8^ cm^2^ s^−1^), indicating a significant enhancement in the diffusion kinetics of the composite.

As shown in Figure 9d, electrochemical impedance spectroscopy (EIS) measurements were conducted to further confirm the contribution of the carbon amount (6.31 wt%) to the electrochemical performance. The EIS data were fitted utilizing an embedded equivalent circuit model. Both Nyquist plots show a high-middle frequency semicircle (charge-transfer resistance from the electrochemical charge-transfer process) and a low-frequency straight line. The straight line can be ascribed to the Warburg impedance (W), which correlates with the diffusion behavior of Li^+^ within the active material [34]. As shown in Figure 9d, the semicircle diameter exhibited by the LTO/C-6.31 wt% composite material is markedly smaller than that of the pure LTO material in the high–middle frequency range, signifying a reduced charge transfer resistance in the composite. In the low frequency range, the slope of LTO/C-6.31 wt% composite material surpasses that of LTO, indicating an accelerated diffusion rate of lithium ions within the composite. Quantitatively, the R_s_ and R_ct_ for LTO/C-6.31 wt% composite are 3.978 Ω and 11.76 Ω, respectively, which are remarkably lower than those of pure LTO (5.452 Ω, 45.9 Ω), exhibiting faster electrochemical dynamics. The above EIS results align closely with the rate performance test results.

**Table 1:** Electrochemical performance comparison of LTO/C-6.31 wt% composite with other reported LTO composites materials.

Materials	Carbon Content (wt%)	Current Rate (C)	Rate Capacity (mAh g^−1^)	Current Rate for Cycling (C)	Cycle Number	Capacity Retention (%)	References
LTO/C	6.31	1, 20	181.5, 154.9	20	1000	93.93	This work
LTO/C	3.74	1, 20	174.1, 147.4	20	1000	80.5	[35]
LTO/C	5	0.1, 20	165.5, 147.7	20	200	92.6	[26]
LTO/C/SG	5.3	0.1, 10	171.9, 157.2	20	800	96.2	[36]
C/N/S-LTO	/	1, 20	167.3, 143.7	20	1000	94	[37]
Co/S-LTO	/	1,20	174.1,158.6	20	1000	85.3	[38]

## 4. Conclusions

In conclusion, pringle-shaped amorphous Li_4_Ti_5_O_12_/carbon (LTO/C) composites were synthesized using low-cost sucrose as the organic carbon source, via a facile hydrothermal-calcination method. Three weight ratios of carbon coating (4.95 wt%, 6.31 wt% and 8.65 wt%) were prepared and investigated to find the optimal carbon among the three designed carbon content samples in this work. Electrochemical characterization showed that the LTO/C-6.31 wt% composite exhibited outstanding rate capability and cycling stability (145.5 mAh g^−1^ at 20 C after 1000 cycles, capacity retention of 93.93%). This enhanced electrochemical performance is attributed to three key factors: (i) The interparticle mesopores within the LTO nanocrystallites facilitate rapid electrolyte infiltration and shorten Li+ diffusion channels. (ii) The conductive network constructed by the carbon phase significantly enhances the electronic conduction capability of the electrode material. (iii) The synergistic effect between the LTO and the carbon phase synergistically promotes the charge-transfer reactions. This finding comprehensively demonstrates the exceptional potential of the LTO/C composite material for high-power energy storage applications, and offers a relatively innovative design strategy for the development of anode materials tailored to fast-charging lithium-ion batteries.

## Data Availability

The original contributions presented in this study are included in the article. Further inquiries can be directed to the corresponding authors.
